# The effect of Area Deprivation Index on clinical outcomes after total shoulder arthroplasty

**DOI:** 10.1016/j.jsea.2026.100014

**Published:** 2026-03-20

**Authors:** Jared J. Reid, Trevor D. Wolterink, Robert J. Reis, Brandon L. Rogalski, Josef K. Eichinger, Richard J. Friedman

**Affiliations:** Medical University of South Carolina, Charleston, SC, USA

**Keywords:** Shoulder, Arthroplasty, Outcomes, Area deprivation index, Socioeconomic, Social determinates of health

## Abstract

**Background:**

Socioeconomic status (SES), as measured by the Area Deprivation Index (ADI), is associated with differences in pre-operative status and post-operative outcomes in patients undergoing total shoulder arthroplasty (TSA). The effect of SES on TSA outcomes remains unclear, as studies thus far report mixed findings overall. The purpose of this study is to determine the association between SES at the neighborhood level, using ADI scores, and patient-reported outcome measures (PROMs) following TSA.

**Methods:**

A retrospective review of a prospectively collected single-institution database was performed to identify patients who underwent primary TSA (Advita Ortho) with a minimum 2-year follow-up. Patient addresses were collected and converted to ADI scores. Patients were divided into quartiles of ADI, and the most deprived (ADI_High_) and least deprived (ADI_Low_) quartiles were compared. Pre- and post-operative range of motion (ROM) and PROM, along with complication and revision rates at the last follow-up, were obtained. For ROM, visual analog scale pain, global function score and American Shoulder and Elbow Surgeons scores, the Minimal Clinically Important Difference (MCID), substantial clinical benefit (SCB), and patient acceptable symptom state (PASS) were defined by previously established threshold values. Multivariable logistic regression models were estimated to characterize the association of the ADI with MCID, SCB, and PASS attainment along PROM domains.

**Results:**

A total of 599 procedures with a minimum of 2-year follow-up and ADI score were grouped into distinct quartiles for analysis, and the ADI_Low_ (n = 150, ADI 0-25) and ADI_High_ (n = 150, ADI 67-100) groups were compared. ADI_High_ demonstrated inferior pre-operative ROM and PROM compared to ADI_Low_. These differences decreased post-operatively, remaining statistically but not clinically significant for visual analog scale pain, global shoulder function, and American Shoulder and Elbow Surgeons scores. Each group achieved MCID, SCB, and PASS at similar rates. ADI was not a significant predictor of achieving MCID, SCB, and PASS thresholds.

**Conclusion:**

SES impacts pre-operative disease severity and absolute post-operative outcomes after TSA but does not significantly limit the capacity for clinically meaningful improvement. Disadvantaged patients derive similarly substantial benefit from surgery compared to those from higher SES backgrounds.

Social determinants of health (SDOH), defined by the World Health Organization as the conditions in which people are born, grow, live, work, and age, are increasingly utilized as predictors of post-operative outcomes.^7^, ^8^, ^9^, ^10^, ^11^ Though innumerable factors comprise SDOH, socioeconomic status (SES) is a substantial component, determined by income, education level, occupation, living conditions, and access to resources.^39^ SES is a known predictor of morbidity and mortality among orthopedic subspecialties, closely intertwined with the quality of care provided.^39^

Traditionally, insurance status has been utilized as a proxy for SES, with patient-related factors less readily available from medical records. However, the Area Deprivation Index (ADI) has enabled quantification of SES, providing a composite neighborhood-based indicator of socioeconomic disadvantage.^6^ The national ADI provides normalized scores based on 17 indicators from the U.S. Census data, ranging from 1 to 100. Higher scores indicate greater social deprivation or disadvantage, and lower scores indicate less social deprivation or advantage.

Limited studies have examined the impact of ADI on outcomes following total shoulder arthroplasty (TSA) with mixed results overall.^17^^,^^19^^,^^23^ Therefore, the purpose of this study is to determine the association between SES at the neighborhood level, using ADI scores, and patient-reported outcome measures (PROM) following TSA. We hypothesize that individuals residing in lower SES neighborhoods, indicated by higher ADI scores, will demonstrate lower PROM overall with less clinical improvement compared to individuals living in high SES neighborhoods, indicated by lower ADI scores.

## Methods

### Study population

With institutional review board approval, a retrospective review of a prospectively collected single-institution shoulder arthroplasty database with 2 fellowship-trained shoulder and elbow surgeons was performed (Advita Ortho, Gainesville, FL, USA). We identified 599 patients who underwent primary anatomic and reverse TSA between January 2012 and February 2022 with a minimum of a 2-year follow-up. Exclusion criteria included diagnoses of fracture, tumor, or infection as well as patients without an identifiable ADI score. Patient home addresses were utilized to determine corresponding national ADI score based on the 2023 ADI dataset.

Patients were then divided into quartiles of ADI, such that ADI values 0 to 25 were included in the first quartile, and 67 to 100 in the fourth quartile, constituting ADI_Low_ (n = 150) and ADI_High_ (n = 150) groups, respectively. These groups were used for subsequent analyses, performed similarly to previous orthopedic literature assessing post-operative outcomes.^5^^,^^26^^,^^31^^,^^32^^,^^42^

### Area Deprivation Index

ADI is a composite score that ranks neighborhoods based on their level of socioeconomic disadvantage. It is calculated using publicly available data, combining 17 indicators across 4 primary domains: income, education, employment, and housing quality. These indicators are statistically combined to create a single score for a specific geographic area, allowing policies and interventions to be targeted at areas with greatest need. A higher ADI score indicates greater deprivation, while a lower score indicates less deprivation. ADI is considered one of the most scientifically validated indices for assessing SDOH.^15^^,^^20^

### Clinical outcomes

Clinical outcomes including shoulder range of motion (ROM), visual analog scale (VAS) pain, global shoulder function, and American Shoulder and Elbow Surgeons (ASES) scores were evaluated at pre- and the latest post-operative clinic visits.^25^ ROM was assessed at clinical visits using a handheld goniometer by the operative surgeon or a trained research coordinator. ROM parameters included forward elevation (FE), internal rotation (IR) score, and external rotation (ER). FE and ER were measured in degrees, and IR score was determined by the most cephalad vertebral level reached by the thumb behind the patient's back.^37^

### Statistical analysis

Data analysis was conducted using IBM SPSS Statistics 28.0 (Armonk, NY, USA) and Microsoft Excel 2019 (Redmond, WA, USA). Continuous variables were analyzed by an independent sample *t*-test, and categorical variables were analyzed by a chi-square test. Achievement of the Minimal Clinically Important Difference (MCID), substantial clinical benefit (SCB), and patient acceptable symptom state (PASS) for PROM and clinical outcomes were determined based on thresholds from Simovitch et al.^35^ Binomial logistic regression was performed using all patients (n = 599) to assess the association of ADI with MCID, SCB, and PASS attainment along PROM domains. Covariates controlled for during analysis include all variables found in Tables I and II. For all statistical comparisons, an alpha value, *P* < .05, defined statistical significance.

## Results

There were 150 patients included in each of the ADI_Low_ and ADI_High_ cohorts. Demographics were similar between groups (Table I). The ADI_Low_ cohort was composed of a higher proportion of white patients (88% vs. 68%, *P* < .001) while the ADI_High_ cohort was composed of a higher proportion of black patients (11% vs. 32%, *P* < .001). The ADI_High_ cohort demonstrated an increased rate of diabetes mellitus (7% vs. 25%, *P* < .001) and significantly fewer patients reporting no comorbid conditions at all (36% vs. 25%, *P* = .033). Similar complication (3% vs. 3%, *P* = .735) and revision rates (3% vs. 1%, *P* = .409) were observed between groups.

**Table I:** Comparison of peri-operative categorical variables between ADI_Low_ and ADI_High_ patient cohorts.

	ADI_Low_ (N = 150)	ADI_High_ (N = 150)	*P* value
Variable	%	%	
Female sex	53	61	.130
Race			
White	88	68	**<.001**
Black	11	32	**<.001**
Other	1	0	.317
Surgery on dominant arm	60	53	.200
Previous surgery	27	28	.897
Underwent aTSA	28	21	.180
Pre-operative diagnosis			
Osteoarthritis	87	79	.063
Rotator cuff tear	24	32	.123
Rotator cuff arthropathy	21	20	.886
Comorbidities			
None	36	25	**.033**
Hypertension	52	63	.062
Heart disease	13	15	.739
Diabetes	7	25	**<.001**
Tobacco use	6	9	.278
Chronic renal failure	2	3	.474
Other	5	3	.556
Complication	3	3	.735
Revision	3	1	.409

*ADI*, Area Deprivation Index; *aTSA*, anatomic total shoulder arthroplasty.

Bold indicates statistically significant, *P* < .5.

Pre-operative ROM was significantly greater in the ADI_Low_ cohort compared to the ADI_High_ cohort in all planes measured (Table II). Pre-operative mean and worst VAS scores were decreased in the ADI_Low_ cohort at 5.7 and 8.4 compared to the ADI_High_ cohort at 7.2 (*P* < .001) and 9.5 (*P* < .001). Pre-operative PROMs, measured as global shoulder function and ASES, were increased in the ADI_Low_ cohort at 4.8 and 40 compared to the ADI_High_ cohort at 4.1 (*P* = .014) and 26 (*P* < .001). The ADI_High_ cohort had a higher mean body mass index (29 vs. 31, *P* < .001).

**Table II:** Comparison of continuous peri-operative variables between ADI_Low_ and ADI_High_ patient cohorts.

	ADI_Low_	ADI_High_	*P* value
Variable	Mean	Mean	
Duration of follow-up (yr)	4.2	4.2	.955
Age	69	67	.052
BMI (kg/m^2^)	29	31	**<.001**
Pre-operative			
Active FE (°)	118	101	**<.001**
Active IR score	4.7	4.2	**.011**
Active ER (°)	35	28	**.006**
VAS average pain	5.7	7.2	**<.001**
VAS worst pain	8.4	9.5	**<.001**
Global shoulder function	4.8	4.1	**.014**
ASES score	40	27	**<.001**
Post-operative			
Active FE (°)	155	148	**.036**
Active IR score	5.5	5.2	.125
Active ER (°)	55	54	.557
VAS average pain	1	1.4	.066
VAS worst pain	2.2	3.3	**.003**
Global shoulder function	8.7	8.3	**.042**
ASES score	88	80	**<.001**

*ADI*, Area Deprivation Index; *BMI*, body mass index; *FE*, forward elevation; *IR*, internal rotation; *ER*, external rotation; *VAS*, visual analog scale; *ASES*, American Shoulder and Elbow Surgeons.

Bold indicates statistically significant, *P* < .5.

Post-operatively, active FE remained significantly greater in the ADI_Low_ cohort (155 vs. 148, *P* = .036), but differences in active IR (5.5 vs. 5.2, *P* = .125) and active ER (55 vs. 54, *P* = .557) were no longer statistically significant (Table II). VAS average pain was similar between cohorts post-operatively, but VAS worst pain scores were significantly increased (2.2 vs. 3.3, *P* = .003) in the ADI_High_ cohort. Regarding PROM, significantly greater post-operative global shoulder function (8.7 vs. 8.3, *P* = .042) and ASES score (88 vs. 80, *P* < .001) were achieved in the ADI_Low_ cohort (Table II). However, none of these statistically significant differences post-operatively between cohorts exceeded the MCID threshold criteria established by Simovitch et al^35^

Overall, the majority of patients in both the ADI_Low_ and ADI_High_ cohorts achieved MCID, SCB, and PASS thresholds across all variables (Table III). The ADI_Low_ cohort demonstrated a decreased rate of SCB attainment for FE (53% vs. 66%, *P* = .047) and a decreased rate of MCID and SCB attainment for VAS pain (79% vs. 88%, *P* = .035 for MCID; 61.4% vs. 78.1%, *P* = .003 for SCB). The ADI_Low_ cohort demonstrated an increased rate of PASS attainment for ASES (79% vs. 66%, *P* = .020). Binomial logistic regression, including patients from the entire study cohort (n = 599), did not demonstrate a significant association between ADI and attainment of MCID, SCB, or PASS.

**Table III:** Proportion achieving MCID, SCB, and PASS thresholds at final follow-up in ADI_Low_ and ADI_High_ patient cohorts.

	ADI_Low_	ADI_High_	*P* value
Variable	%	%	
MCID			
Active FE (°)	71	75	.397
Active IR score	50	58	.216
Active ER (°)	74	81	.167
VAS pain	79	88	**.035**
Global function score	81	84	.479
ASES score	93	95	.543
SCB			
Active FE (°)	53	66	**.047**
Active IR score	41	53	.067
Active ER (°)	71	81	.053
VAS pain	61	78	**.003**
Global function score	69	72	.531
ASES score	78	82	.426
PASS			
Active FE (°)	85	81	.429
Active IR score	98	94	.179
Active ER (°)	94	94	.840
VAS pain	71	64	.249
Global function score	89	82	.102
ASES score	79	66	**.020**

*ADI*, Area Deprivation Index; *FE*, forward elevation; *IR*, internal rotation; *ER*, external rotation; *VAS*, visual analog scale; *ASES*, American Shoulder and Elbow Surgeons; *MCID*, Minimal Clinically Important Difference; *SCB*, substantial clinical benefit; *PASS*, patient acceptable symptoms state.

Bold indicates statistically significant, *P* < .5.

## Discussion

Our findings demonstrate inferior baseline ROM, VAS pain, and shoulder function in socioeconomically disadvantaged patients (ADI_High_) compared to socioeconomically advantaged patients (ADI_Low_) undergoing primary TSA. However, each group demonstrated clinically indistinguishable improvement across all variables, independent of ADI.

With the emergence of ADI, a more precise portrayal of an individual's socioeconomic environment can be leveraged to elucidate the impact of SDOH on health-related metrics.^6^ Prior studies have relied upon single variables, such as insurance type, to exclusively represent SES. Conversely, ADI is a composite index, providing a more holistic measure of neighborhood deprivation.^20^ As a result, the ADI has gained substantial traction within the literature and has shown to affect post-operative outcomes across multiple orthopedic procedures.^14^^,^^18^^,^^31^^,^^36^^,^^41^

To date, few studies have examined the impact of ADI on outcomes following TSA, with mixed results overall.^17^^,^^19^^,^^23^ These studies have consistently reported increased pain and inferior shoulder function among socioeconomically disadvantaged patients, indicated by higher ADI scores, corroborated by our findings here.^19^^,^^23^^,^^24^^,^^34^ This finding among socioeconomically disadvantaged patients has previously been attributed to increased disease burden at the time of presentation, barriers to high-quality musculoskeletal care, manual or repetitive-labor occupations, comorbid conditions and multiple biopsychosocial factors.^13^^,^^27^^,^^28^^,^^40^

This study does not provide an objective radiographic measure of the severity of glenohumeral osteoarthritis to further quantify and correlate disease burden at the time of presentation to subjective debilitation. However, the ADI_High_ group was composed of a significantly greater proportion of minority patients, consistent with prior reports of racial composition within socioeconomically disadvantaged populations in the United States.^3^^,^^30^^,^^38^ Moreover, these findings reiterate the prevailing theme of white, socially nondeprived males with private insurance undergoing orthopedic procedures at relatively increased rates, highlighting deficiencies in the provision of equitable orthopedic care across all populations.^30^ In addition to this, significantly higher rates of diabetes mellitus were reported in the ADI_High_ group, along with significantly fewer patients reporting no comorbid conditions at all, consistent with previous reports of higher comorbidity rates among individuals with lower SES.^1^^,^^22^^,^^29^ As for biopsychosocial factors, chronic economic stress, job insecurity, and housing or food instability impart increased psychosocial stress linked to greater pain sensitivity across multiple body regions, including the shoulder, likely contributing to increased pre-operative pain observed in the ADI_High_ group here.^12^

Complication and revision rates were similar between ADI_High_ and ADI_Low_ groups in this study. This finding contradicts a previously published study that found positive associations with increasing social deprivation and increasing hospital readmission, emergency department visits, dislocation rate, and medical complications, especially in the short-term post-operative period.^2^ A separate propensity-matched analysis of 8,023 patients undergoing TSA additionally observed patients with increasing social deprivation at greater risk of 90-day medical complications.^33^ Additionally, a large retrospective review of 17,698 patients undergoing primary TSA observed higher rates of humeral fracture and dislocation in patients with increased social deprivation.^21^ It may be that this study, with a smaller cohort, was unable to detect differences in relatively low complication and revision rates compared to these large, propensity-matched database reviews.^2^^,^^33^

Though statistically and clinically significant differences were observed pre-operatively, no clinically significant differences between ADI_High_ and ADI_Low_ groups were observed post-operatively. Even further, each group, regardless of ADI, achieved MCID, SCB, and PASS thresholds at similar rates. Prior studies have reported mixed findings regarding the expected clinical improvement following TSA in socioeconomically disadvantaged groups, based on ADI.^4^^,^^16^^,^^19^^,^^23^^,^^24^^,^^34^ Morgan et al observed 170 patients undergoing primary TSA, reporting similar rates of clinical improvement between groups following surgery compared to baseline scores, regardless of ADI.^23^ These findings, in concert with our own, suggest TSA remains a successful operation, regardless of SES.

However, these findings are contradicted by Khlopas et al, who found a weak association between decreased ADI and inferior function pre- and post-operatively as well as pre- to post-operative change.^17^ However, no difference in the proportion of each ADI group achieving MCID, SCB, and PASS following anatomic TSA was found, as observed in our study. This finding suggests that the weak negative correlation between ADI and functional outcome measures reported by Khlopas et al is statistically significant but clinically irrelevant.^19^ Khlopas et al did preform a separate subanalysis of reverse total shoulder arthroplasty (rTSA) alone, which yielded decreased rates of MCID, SCB, and PASS threshold achievement with increased ADI, yet our study does not offer a similar, stratified analysis for comparison. Lastly, findings from Moverman et al differ from those discussed thus far and found equivalent pre- and post-operative outcomes, regardless of ADI.^24^ This finding of equivalent pre-operative baseline function between ADI groups contradicts the literature, with the authors attributing this to either type II sampling error or inability to determine if their respective socioeconomically disadvantaged cohort had presented earlier than in similar cohorts observed by other studies.

Following logistic regression analysis, ADI was not a significant predictor of achieving MCID, SCB, and PASS thresholds. Of note, both ADI_High_ and ADI_Low_ groups were composed of varying proportions of anatomic TSA and rTSA. To control for this, analysis was performed by using MCID, SCB, and PASS thresholds specific for each type of TSA, including the type of TSA as a covariate.

This study provides valuable insight from a distinct geographic cohort utilizing ADI, one of the most scientifically validated indices for assessing SDOH. Still, our study is not without limitations. This work was conducted at a single academic institution in the Southeast, which may not reflect the national population, making generalizability of our findings difficult. Furthermore, ADI is utilized as an improved, composite proxy index of SES, yet all socioeconomic challenges and barriers to healthcare access faced by patients are not captured. Additionally, ADI is an area-level measure, dominated by few housing and income variables which may lead to misclassification of individuals, emphasizing the need for patient-level data to provide more nuanced structural measures. Regarding our analysis, the top and bottom quartiles, based on ADI, were examined with similar studies performed previously. With this, inherent selection bias following omission of the middle quartiles may distort estimated associations observed, yet given our findings of no clinically distinguishable differences post-operatively when comparing extremes, inclusion of the middle quartiles during analysis would likely have no effect on the conclusions determined. However, the regression analysis did include all patient quartiles and supported observations found with comparison of the top and bottom quartiles. Additionally, comparison of the extremes did weaken our ability to detect small effect sizes, increasing susceptibility to type II error, yet high-quality, prospectively collected outcomes enhance the validity of estimates from a smaller sample and can be more informative than a larger but heterogeneous registry-type cohort. Despite these limitations, this study contributes further to the growing body of literature investigating the influence of SES on outcomes following TSA.

## Conclusions

SES impacts pre-operative disease severity and absolute post-operative outcomes after TSA but does not significantly limit the capacity for clinically meaningful improvement. Disadvantaged patients derive similarly substantial benefit from surgery compared to those from higher SES backgrounds.

## Disclaimers:

Funding: No funding was disclosed by the authors.

Conflicts of interest: Josef K. Eichinger reports grants and nonfinancial support from Advita Ortho and received personal fees from FH Ortho outside the submitted work. Richard J Friedman reports grants and personal fees from Advita Ortho. Any additional authors, their immediate families, and any research foundations with which they are affiliated have not received any financial payments or other benefits from any commercial entity related to the subject of this article.
